# Cumulative occupational lumbar load and lumbar disc disease – results of a German multi-center case-control study (EPILIFT)

**DOI:** 10.1186/1471-2474-10-48

**Published:** 2009-05-07

**Authors:** Andreas Seidler, Annekatrin Bergmann, Matthias Jäger, Rolf Ellegast, Dirk Ditchen, Gine Elsner, Joachim Grifka, Johannes Haerting, Friedrich Hofmann, Oliver Linhardt, Alwin Luttmann, Martina Michaelis, Gabriela Petereit-Haack, Barbara Schumann, Ulrich Bolm-Audorff

**Affiliations:** 1 Federal Institute for Occupational Safety and Health, Nöldnerstraße 40–42, 10317 Berlin, Germany; 2 Institute for Medical Epidemiology, Biostatistics, and Informatics, Martin Luther University Halle-Wittenberg, Magdeburger Str. 8, 06097 Halle/Saale, Germany; 3 Institute for Occupational Physiology at the University of Dortmund, Ardeystraße 67, 44139 Dortmund, Germany; 4 Institute for Occupational Safety and Health, BGIA, German Statutory Accident Insurance, Alte Heerstraße 111, 53757 Sankt Augustin, Germany; 5 Institute of Occupational Medicine, Frankfurt University, Johann Wolfgang Goethe-Universität, Theodor Stern-Kai 7, 60590 Frankfurt am Main, Germany; 6 Orthopaedic Clinic, University of Regensburg, Kaiser Karl V Allee 3, 93077 Bad Abbach, Germany; 7 Research Center for Occupational and Social Medicine Freiburg, Bertoldstraße 27, 79098 Freiburg, Germany; 8 Labor inspection, Occupational Health Division, Regional Government of South Hesse, Simone Veil Straße 5, 65197 Wiesbaden, Germany

## Abstract

**Background:**

The to date evidence for a dose-response relationship between physical workload and the development of lumbar disc diseases is limited. We therefore investigated the possible etiologic relevance of cumulative occupational lumbar load to lumbar disc diseases in a multi-center case-control study.

**Methods:**

In four study regions in Germany (Frankfurt/Main, Freiburg, Halle/Saale, Regensburg), patients seeking medical care for pain associated with clinically and radiologically verified lumbar disc herniation (286 males, 278 females) or symptomatic lumbar disc narrowing (145 males, 206 females) were prospectively recruited. Population control subjects (453 males and 448 females) were drawn from the regional population registers. Cases and control subjects were between 25 and 70 years of age. In a structured personal interview, a complete occupational history was elicited to identify subjects with certain minimum workloads. On the basis of job task-specific supplementary surveys performed by technical experts, the situational lumbar load represented by the compressive force at the lumbosacral disc was determined via biomechanical model calculations for any working situation with object handling and load-intensive postures during the total working life. For this analysis, all manual handling of objects of about 5 kilograms or more and postures with trunk inclination of 20 degrees or more are included in the calculation of cumulative lumbar load. Confounder selection was based on biologic plausibility and on the change-in-estimate criterion. Odds ratios (OR) and 95% confidence intervals (CI) were calculated separately for men and women using unconditional logistic regression analysis, adjusted for age, region, and unemployment as major life event (in males) or psychosocial strain at work (in females), respectively. To further elucidate the contribution of past physical workload to the development of lumbar disc diseases, we performed lag-time analyses.

**Results:**

We found a positive dose-response relationship between cumulative occupational lumbar load and lumbar disc herniation as well as lumbar disc narrowing among men and women. Even past lumbar load seems to contribute to the risk of lumbar disc disease.

**Conclusion:**

According to our study, cumulative physical workload is related to lumbar disc diseases among men and women.

## Background

Several epidemiological studies show a relationship between lumbar disc diseases and physical workplace factors such as lifting or carrying of loads, forward bending, and whole body vibrations [1-16]; cf. for review, [17]. In some countries as Germany, France, and Denmark, it was decided to include lumbar disc diseases in the list of occupational diseases. However, recognition criteria differ considerably in the mentioned countries with respect to the required occupational exposures and with respect to the required clinical diagnosis. In Germany, the occupational disease No. 2108 is defined as follows: "Disc-related diseases of the lumbar spine caused by the lifting or carrying of heavy loads over many years or by performance of work in an extremely bent posture over many years which have forced the person to discontinue all activities that caused or could cause the onset, worsening or recurrence of the disease". In Denmark, according to the "List of Occupational Diseases Reported on or after January 1, 2005" , the occupational disease No. B.1. is defined as follows: "Chronic low-back disease with pain *(lumbago/sciatica, lumbar prolapsed disc, degenerative low-back disease)"*. In Denmark, the following exposures are required for recognition as an occupational disease: (a) Back-loading lifting work involving lifting/upward pulling of heavy objects and many tonnes of lifting per day for a considerable number of years; (b) Back-loading lifting work with generally occurring, extremely heavy and awkward single lifts and several tonnes of lifting per day for a considerable number of years; (c) Back-loading care work with many daily handlings of adults or older handicapped children for a considerable number of years; (d) Back-loading, daily exposure to whole-body vibrations from heavily vibrating vehicles for a considerable number of years. In France, according to table ("tableau") 98 only lumbar disc herniations associated with radicular symptoms can be recognized as an occupational disease . It is a precondition that heavy objects have been handled for at least five years in specified occupations.

The considerable differences in recognition criteria might at least partly reflect the to date limited evidence for a dose-response relationship between physical workload and the development of lumbar disc diseases. We therefore conducted a population-based case-control study that deals with the following questions:

- Does a positive dose-response relationship exist between occupational lumbar load and lumbar disc diseases (lumbar disc herniation or lumbar disc narrowing)?

- Which type of exposure does contribute to the lumbar disc disease risk [i.e., working situations with increased lumbar load through holding or moving objects in diverse postures ("manual materials handling") and/or through postures without object handling ("intensive-load postures")]?

- Does past physical work play an etiologic role in the development of lumbar disc disease (in other words: Is a cumulative lumbar-load dose model appropriate)?

## Methods

### Study population

Subject recruitment in our study was performed prospectively in four study regions in Germany: Frankfurt am Main, Freiburg, Halle/Saale, and Regensburg. In the mentioned regions, all hospitals or practices (n = 29) treating at least five patients with lumbar disc herniation per year as well as a random sample of orthopedic practices (treating patients with lumbar disc narrowing; n = 14) were included. The corresponding physicians were asked to identify all patients between 25 and 70 years that belonged to one of the following case groups:

Case group 1

Males with outpatient or inpatient treatment in an included hospital because of lumbar disc herniation with sensitive and/or motor radix syndrome;

Case group 2

Females with lumbar disc herniation as defined under case group 1;

Case group 3

Males with outpatient or inpatient treatment in a hospital or outpatient treatment in an included orthopedic practice due to severe disc narrowing (of more than one third compared with adjacent unchanged discs) with sensitive and/or motor radix syndrome or with local lumbar syndrome with a finger floor distance of more than 25 cm;

Case group 4

Females with lumbar disc narrowing as defined under case group 3.

The diagnosis of lumbar disc herniation had to have been confirmed by computerised tomography (CT) or by magnetic resonance imaging (MRI); the diagnosis of lumbar disc narrowing was primarily based on X-ray. Disc herniation and disc narrowing were defined as proposed by a German consensus group [18]. Altogether, 1,112 patients were reported by the participating physicians. To finally qualify as cases, MRI, CT and X-rays of the lumbar spine were re-assessed by one reference radiologist separately for each disc and vertebral body. Furthermore, the clinical diagnosis had to be verified by one experienced reference orthopedist (O.L.). As a consequence of this diagnostic verification, 197 patients did not fulfill the inclusion criteria and were excluded from the study. In total, 915 cases were included: 286 males (case group 1) and 278 females (case group 2) with lumbar disc herniation, 145 males (case group 3) and 206 females (case group 4) with lumbar disc space narrowing. About half of the cases with lumbar disc herniation showed a motor or sensomotor radix syndrome (55% of male, 49% of female patients, see table 1). Of male cases with symptomatic lumbar disc narrowing, 31% had a motor or sensomotor radix syndrome, 23% had a sensitive radix syndrome, and 46% solely had a local lumbar syndrome. Of female cases with symptomatic lumbar disc narrowing, 25% had a motor or sensomotor radix syndrome, 19% had a sensitive radix syndrome, and 54% solely had a local lumbar syndrome. The participation rate was 66.4% among the cases.

**Table 1:** Characteristics of the cases with lumbar disc disease and control subjects separately for men and women

	Males						Females					
		
	Lumbar disc herniation (n = 286)		Lumbar disc narrowing (n = 145)		Control subjects (n = 453)		Lumbar disc herniation (n = 278)		Lumbar disc narrowing (n = 206)		Control subjects (n = 448)	
	N	%	N	%	N	%	N	%	N	%	N	%
Clinical symptoms												
Motor/sensomotor radix syndrome	157	54.9	43	31.0			137	49.3	52	25.2		
Sensitive (and no motoric) radix syndrome	128	44.8	33	22.8			138	49.6	40	19.4		
Motor and/or sensitive radix syndrome, not further classified	1	0.3	1	0.7			3	1.1	2	1.0		
Local lumbar syndrome	-	-	66	55.5			-	-	112	54.4		

Age at diagnosis/interview												
< 35 years	27	9.4	9	6.2	84	18.5	51	18.3	4	1.9	77	17.2
35 – <45 years	91	31.8	20	13.8	119	26.3	71	25.5	27	13.1	137	30.6
45 – <55 years	73	25.5	31	21.4	97	21.4	72	25.9	54	26.2	109	24.3
55 – <65 years	61	21.3	51	35.2	104	23.0	58	20.9	78	37.9	94	21.0
> = 65 years	34	11.9	34	23.4	49	10.8	26	9.4	43	20.9	31	6.9
*Mean (SD)*	*48.7 (11.1)*		*55.0 (10.7)*		*47.3 (12.6)*		*47.1 (11.8)*		*56.0 (9.8)*		*46.4 (11.8)*	

Region												
Frankfurt am Main	74	25.9	46	31.7	108	23.8	69	24.8	39	18.9	105	23.4
Freiburg	75	26.2	35	24.1	122	26.9	75	27.0	51	24.8	115	25.7
Halle/Saale	81	28.3	36	24.8	104	23.0	75	27.0	66	32.0	117	26.1
Regensburg	56	19.6	28	19.3	119	26.3	59	21.2	50	24.3	111	24.8

Educational level												
Graduated from high school	74	25.9	26	17.9	182	40.2	64	23.0	27	13.1	145	32.4
Secondary school level	83	29.0	32	22.1	125	27.6	101	36.3	59	28.6	173	38.6
Elementary level/no graduation	127	44.7	87	60.0	146	32.2	112	40.3	119	57.8	129	28.8
Unknown	2	0.7	-	-	-	-	1	0.4	1	0.5	1	0.2

Unemployment/dismissal^a^												
No	236	82.5	121	83.4	335	74.0	240	86.3	154	74.8	355	79.2
Yes, little strain	12	4.2	3	2.1	21	4.6	6	2.2	4	1.9	14	3.1
Yes, medium strain	13	4.5	3	2.1	27	6.0	7	2.5	8	3.9	27	6.0
Yes, high strain	12	4.2	10	6.9	44	9.7	10	3.6	16	7.8	32	7.1
Yes, very high strain	12	4.2	8	5.5	24	5.3	15	5.4	23	11.2	20	4.5
Unknown	1	0.3	-	-	2	0.4	-	-	1	0.5	-	-

Psychosocial workload (job strain)												
1^st ^tertile	63	22.0	41	28.3	143	31.6	77	27.7	48	23.3	148	33.0
2^nd ^tertile	108	37.8	52	35.9	159	35.1	72	25.9	45	21.8	129	28.8
3^rd ^tertile	93	32.5	35	24.1	123	27.2	101	36.3	77	37.4	119	26.6
High job strain (95%-percentile)	19	6.6	14	9.7	19	4.2	23	8.3	32	15.5	26	5.8
Not applicable (e.g., self-employed persons)	-	-	-	-	3	0.7	-	-	-	-	15	3.3
Unknown	3	1.0	3	2.1	6	1.3	5	1.8	4	1.9	11	2.5

^a ^Distress related to critical life event.

Control subjects were randomly selected from a one percent random sample of residents aged 25 to 70 years drawn by the local population registration offices of the respective region. Of 1,687 population controls, 901 agreed to participate (53.4%). According to a non-responder analysis, the proportion of blue-collar workers was higher among non-responding cases as well as among non-responding control subjects. We found no evidence for a differential selection bias with respect to social status.

### Standardized personal interview

The interviewers documented a complete (self-reported) occupational history for each participant, including every occupational period that lasted at least half a year. Interviewers were intensively trained in standardized interview techniques and a non-differential approach to cases and controls. Participants were not informed of the specific aims of the study. Actually, they were asked to participate in a study concerning the theme 'occupation and health'. A detailed computer-assisted personal interview was developed to elicit information about psychosocial workload (German screening tool "FIT", based on Karasek's job strain model)[19,20]; leisure activities; and level of distress through critical life events. All subjects also answered the Nordic questionnaire on musculoskeletal symptoms [21]. Occupational physical activities including lifting and carrying of objects, pushing and pulling, working in trunk-flexed postures, and whole-body vibration were registered in order to identify subjects with certain minimum workloads.

### Expert assessment of occupational exposure, biomechanical analysis and choose of dose model

With those subjects who (on the basis of the standardized personal interview) exceeded relatively low "exposure thresholds" in lifting, carrying, pulling, pushing of objects, working postures or whole-body vibration, subsequently a semi-standardized personal comprehensive expert interview was performed by branch-orientated ergonomic experts of the institutions for statutory accident insurance and prevention. The experts were blinded for the case-control status. On the basis of job-task specific supplementary surveys, these experts documented the intensity, frequency and duration of all spine-related exposures induced by manual materials handling (e.g., lifting, carrying, pushing, pulling, throwing, catching or shoveling of all objects weighing at least 5 kilograms), trunk inclination and twisting, and whole-body vibration for the entire working life of each subject [22].

A biomechanical analysis based on this expert assessment was conducted to determine the "situational lumbar load". As the characteristic indicator of lumbar load, the compressive force on the lumbosacral disc was calculated for each loading activity (range 0 to 8.4 Kilo-Newton). Quantification was based on biomechanical-model calculations applying the three-dimensional dynamic simulation tool "The Dortmunder" [23].

Comprehensive analyses were done to identify the "best" dose model (for a brief description of the ten analysed dose models, please see Appendix 1) in terms of goodness of fit (estimated by the Akaike information criterion AIC), specifity, simplicity, and clarity of the dose-response relationship. First results of these analyses have been published elsewhere [24,25]. However, the question of the "best" dose model is of minor importance to the appropriateness of the cumulative dose concept. Therefore, in this analysis all biomechanically relevant loading actions which are assumed to potentially contribute to the development of lumbar disc disease are included in the calculation of cumulative lumbar load: For the calculation of cumulative lumbar load manual handling of objects of about 5 kilograms or more and postures with trunk inclination of at least 20 degrees or more are considered. Lumbar-disc compressive force is weighted overproportionally (squared) in relation to the respective duration of materials handling or intensive-load posture.

#### Reproducibility of cumulative occupational lumbar load

To evaluate the reliability of cumulative occupational lumbar load calculated on the basis of expert assessments, 40 expert interviews and subsequent expert assessments were repeated by the same experts (intra-rater reliability), and further 40 expert interviews and subsequent expert assessments were repeated by different experts (inter-rater reliability). We calculated Kappa values comparing the categorised cumulative exposures derived from the first expert interview with the corresponding exposure categories derived from the retest. Intra-rater agreement (Kappa = 0.64) as well as inter-rater agreement (Kappa = 0.46) were fair to good.

### Categorization of variables

As an a-priori defined procedure, all continuous variables were categorized in tertiles based on the distribution of the exposed control subjects. If less than 20% of the control subjects were "non-exposed", the reference category combined non-exposed subjects and subjects in the first exposure tertile. This was the case for all exposure variables in men, while in women, non-exposed persons and the first tertile remained in different categories.

### Description of potential confounders and statistics

Odds ratios (OR) and 95% confidence intervals (CI) were calculated using unconditional logistic regression analysis. All statistical analyses were adjusted for age and place of residence, referred to as "region" in this text. As age is known to be strongly associated particularly with the occurrence of lumbar disc narrowing [1] and as cases with lumbar disc narrowing were on average older than control subjects, we decided to adjust for age. Age was entered into the logistic regression model in ten-year categories. Region was considered to be a potential confounder because occupational exposures were suspected to differ between regions. For the distribution of potential confounders among cases and control subjects see table 1.

Besides the odds ratios solely adjusted for age and region, odds ratios for the fully adjusted "final model" are given. The final model should comprise all factors that might confound the relationship between cumulative lumbar load and lumbar disc herniation or lumbar disc narrowing, respectively. Therefore, selection of confounders was performed based on a list of biologically plausible factors in two steps: 1. Factors were considered as potential confounders if they were correlated with the cumulative lumbar load (Kendall-Tau>0.1) among control subjects. For men, the following factors were identified: weight (body mass index), Scheuermann's disease, articular gout, and whole-body vibrations. In women, solely whole-body vibrations were correlated with the cumulative lumbar load. 2. Potential confounders were included in the final logistic regression model if they changed the odds ratio of cumulative lumbar load by more than 10% in at least one category. In the final model, the following confounders were included: age, region, distress by unemployment as major life event (solely for case group 1), and psychosocial stress (for case groups 2 and 4). No additional confounders were considered in case group 3. Missing values were analysed as a separate exposure category (results not shown here).

### Lag-time analysis

To further elucidate the contribution of past physical workload to the development of lumbar disc herniation, we performed two lag-time analyses: First, we restricted our analysis to cumulative lumbar load that had occurred up to 10 years prior to diagnosis. Second, we performed a subanalysis for all subjects that were not exposed to materials handling or intensive-load postures during 10 years prior to diagnosis.

### Statistical power of the study

Calculation of the statistical power of the study was based on an expected prevalence of heavy physical work of 21% among the male population and 15% among the female population according to Elsner and Seidler [26]. To detect an odds ratio (OR) of 2.0 with a power of 80% for heavy physical work, we planned to include at least 150 male and 200 female cases and 460 male and 460 female control subjects. With the actually attained 145 male cases in case group 3 and 453 control subjects, an odds ratio of 2.0 for heavy physical work could be detected with a power of 88%; with the actually attained 206 female cases in case group 4 and 448 control subjects, an odds ratio of 2.0 for heavy physical work could be detected with a power of 89%; for case groups 1 and 2 the power is considerably higher. These calculations do not take into account the loss of power through differences in confounding factors. However, as previous studies suggested relatively strong effects of physical workload on the occurrence of lumbar disc disease, our sample size appears rather adequate.

### Ethics

The study was regarded as quality development work within the area of occupational health. The aims, methods, and procedures of the study were agreed by the Hesse Medical Association. The study was performed in compliance with the ethical principles of the Helsinki Declaration. The concept of data protection was developed in cooperation with and agreed by the Hesse Federal Commissioner for Data Protection and Freedom of Information Decline. All subjects gave their informed consent.

## Results

Among men (table 2), there was a positive dose-response relationship between the cumulative lumbar load (through manual materials handling and/or intensive-load postures) and the diagnosis of a lumbar disc herniation. In the middle exposure group, the odds ratio of the final model was 1.7 (95% CI 1.1 to 2.4), in the highest exposure category, it was 3.4 (95% CI 2.2 to 5.0). Similar results were found for the risk of lumbar disc narrowing (OR = 3.2; 95% CI 1.9 to 5.5 in the highest category). Among women (table 3), we also found a positive dose-response relationship for lumbar disc herniation. The odds ratio of the final model was 2.4 (95% CI 1.6 to 3.8) in the second-highest exposure category; in the highest exposure category, the OR did not further increase (OR = 2.3, 95% CI 1.5 to 3.6). Also for lumbar disc narrowing, the highest odds ratio was found in the third category (OR = 2.3, 95% CI 1.3 to 3.9); while in the highest exposure category, the OR was 2.0 (95% 1.2 to 3.2).

**Table 2:** Cumulative lumbar load and lumbar disc disease among men

	Control subjects		Lumbar disc herniation						Lumbar disc narrowing					
	N	%	N	%	Adj. OR^a^	95% CI	Adj. OR^b^	95% CI	N	%	Adj. OR^a^	95% CI	Adj. OR^c^	95% CI
Cumulative lumbar load through manual materials handling and/or intensive-load postures														
0 – <5.0*10^6^Nh	159	35.1	54	18.9	1.0	-	1.0	-	27	18.6	1.0	-		
5.0 – <21.51*10^6^Nh	147	32.5	76	26.6	1.6	1.0–2.4	1.7	1.1–2.7	31	21.4	1.6	0.9–2.8		
>21.51*10^6^Nh	147	32.5	156	54.5	3.2	2.1–4.7	3.4	2.2–5.0	87	60.0	3.2	1.9–5.5		
														
Cumulative lumbar load through manual materials handling														
0 – <2.34*10^6^Nh	163	36.0	58	20.3	1.0	-	1.0	-	27	18.6	1.0	-	1.0	-
2.34 – <8.98*10^6^Nh	145	32.0	77	26.9	1.5	1.0–2.2	1.2	0.7–2.0	39	26.9	1.6	0.9–2.8	1.3	0.7–2.6
> = 8.98*10^6^Nh	145	32.0	151	52.8	2.8	1.9–4.1	2.0	1.2–3.5	79	54.5	2.9	1.7–4.9	2.4	1.2–4.6
														
Cumulative lumbar load through intensive-load postures														
0 Nh	129	28.5	45	15.7	1.0	-	1.0	-	26	17.9	1.0	-	1.0	-
>0 – <4.85*10^6^Nh	108	23.8	45	15.7	1.3	0.8–2.1	1.1	0.6–2.0	25	17.2	1.6	0.9–3.1	1.3	0.6–2.6
4.85 – 14.62 *10^6^Nh	108	23.8	84	29.4	2.3	1.4–3.6	1.7	0.9–3.2	37	25.5	2.0	1.1–3.6	1.4	0.7–2.9
> = 14.62*10^6^Nh	108	23.8	112	39.2	2.9	1.9–4.6	1.9	1.0–3.5	57	39.3	2.5	1.4–4.4	1.4	0.7–2.9
														
Lag-time analysis I: Cumulative lumbar load up to 10 years prior to diagnosis or interview date (in controls) = exposure during last 10 years set to zero														
0 – <5.0*10^6^Nh	210	46.4	69	24.1	1.0	-	1.0	-	34	23.4	1.0	-		
5.0 – <21.51*10^6^Nh	133	29.4	102	35.7	2.3	1.5–3.4	2.5	1.7–3.7	39	26.9	1.9	1.1–3.2		
> = 21.51*10^6^Nh	110	24.3	115	40.2	3.5	2.3–5.4	3.7	2.4–5.7	72	49.7	3.1	1.8–5.3		
														
Lag-time analysis II: Cumulative lumbar load; solely subjects unexposed in the last 10 years prior to diagnosis or interview date (in controls) = subjects exposed in the last 10 years excluded														
0 – <5.0*10^6^Nh	37	36.6	15	23.8	1.0	-	1.0	-	9	22.0	1.0	-		
5.0 – <21.51*10^6^Nh	38	37.6	28	44.4	1.7	0.8–3.9	2.0	0.9–4.7	11	26.8	1.5	0.5–4.5		
> = 21.51*10^6^Nh	26	25.7	20	31.7	1.8	0.7–4.5	2.1	0.8–5.5	21	51.2	2.8	0.9–8.8		

^a ^Adjusted for age and region

^b ^Adjusted for age, region, and unemployment as severe life event; OR for manual materials handling additionally adjusted for intensive-load postures and vice versa

^c ^Adjusted for age and region; OR for manual materials handling additionally adjusted for intensive-load postures and vice versa

**Table 3:** Cumulative lumbar load and lumbar disc disease among women

	Control subjects		Lumbar disc herniation						Lumbar disc narrowing					
	N	%	N	%	Adj. OR^a^	95% CI	Adj. OR^b^	95% CI	N	%	Adj. OR^a^	95% CI	Adj. OR^b^	95% CI
Cumulative lumbar load through manual materials handling and/or intensive-load postures														
0 Nh	195	43.5	71	25.5	1.0	-	1.0	-	55	26.7	1.0	-	1.0	-
>0 – <4.04*10^6^Nh	84	18.8	55	19.8	1.9	1.2–3.0	1.6	1.1–2.7	28	13.6	1.4	0.8–2.5	1.2	0.6–2.1
4.04 – <14.47*10^6^Nh	85	19.0	74	26.6	2.7	1.8–4.2	2.4	1.6–3.8	50	24.3	2.9	1.8–5.0	2.3	1.3–3.9
> = 14.47*10^6^Nh	84	18.8	78	28.1	2.8	1.8–4.2	2.3	1.5–3.6	73	35.4	2.6	1.6–4.2	2.0	1.2–3.2
														
Cumulative lumbar load through manual materials handling														
0 Nh	218	48.7	92	33.1	1.0	-	1.0	-	61	29.6	1.0	-	1.0	-
0 – <1.58*10^6^Nh	76	17.0	46	16.5	1.5	1.0–2.4	0.8	0.4–1.6	20	9.7	1.3	0.7–2.4	1.3	0.5–3.3
1.58 – <9.06*10^6^Nh	77	17.2	70	25.2	2.4	1.6–3.6	1.0	0.5–1.9	62	30.1	3.5	2.1–5.6	3.0	1.3–6.8
> = 9.06*10^6^Nh	77	17.2	70	25.2	2.3	1.5–3.5	0.8	0.4–1.6	63	30.6	2.5	1.6–4.0	1.9	0.8–4.4
														
Cumulative lumbar load through intensive-load postures														
0 Nh	206	46.0	75	27.0	1.0	-	1.0	-	61	29.6	1.0	-	1.0	-
>0 – <2.77*10^6^Nh	80	17.9	52	18.7	1.9	1.2–3.0	1.9	1.0–3.7	24	11.7	1.3	0.7–2.4	0.7	0.3–1.7
2.77 – 8.83 *10^6^Nh	81	18.1	66	23.7	2.5	1.6–3.8	2.4	1.2–4.6	45	21.8	2.2	1.3–3.7	0.8	0.3–1.9
> = 8.83*10^6^Nh	81	18.1	85	30.6	3.2	2.1–4.9	3.2	1.6–6.3	76	36.9	2.8	1.8–4.5	1.1	0.5–2.7
														
Lag-time analysis I: Cumulative lumbar load up to 10 years prior to diagnosis or interview date (in controls) = exposure during last 10 years set to zero														
0 Nh	215	48.0	92	33.1	1.0	-	1.0	-	56	27.2	1.0	-	1.0	-
>0 – <4.04*10^6^Nh	97	21.7	60	21.6	1.5	1.0–2.3	1.4	0.9–2.1	37	18.0	1.6	1.0–2.7	1.4	0.8–2.3
4.04 – <14.47*10^6^Nh	70	15.6	62	22.3	2.5	1.6–3.9	2.2	1.4–3.4	44	21.4	2.9	1.7–5.0	2.2	1.3–3.9
> = 14.47*10^6^Nh	66	14.7	64	23.0	2.5	1.6–3.9	2.2	1.4–3.4	69	33.5	2.9	1.8–4.7	2.2	1.3–3.7
														
Lag-time analysis II: Cumulative lumbar load; solely subjects unexposed in the last 10 years prior to diagnosis or interview date (in controls) = subjects exposed in the last 10 years excluded														
0 Nh	195	75.9	71	64.5	1.0	-	1.0	-	55	53.4	1.0	-	1.0	-
>0 – <4.04*10^6^Nh	25	9.7	12	10.9	1.2	0.6–2.6	1.1	0.5–2.4	15	14.6	1.2	0.6–2.8	1.1	0.5–2.5
4.04 – <14.47*10^6^Nh	18	7.0	14	12.7	2.2	1.0–4.8	1.9	0.9–4.3	19	18.4	2.9	1.3–6.6	2.2	1.0–5.2
> = 14.47*10^6^Nh	19	7.4	13	11.8	1.4	0.6–3.1	1.3	0.6–3.0	14	13.6	1.2	0.5–2.8	1.1	0.4–2.5

^a ^Adjusted for age and region

^b ^Adjusted for age, region, and psychosocial workload; OR for manual materials handling additionally adjusted for intensive-load postures and vice versa

When manual handling of objects and intensive-load postures were separately included in the regression model (see OR^a^; not adjusted for each other), both exposures were strongly associated with lumbar disc herniation as well as with lumbar disc narrowing among men and among women. When manual materials handling and intensive-load postures were adjusted for each other, handling of objects was more strongly associated with lumbar disc narrowing than with lumbar disc herniation (see OR^b ^for men with lumbar disc herniation and for women, OR^c ^for men with lumbar disc narrowing). This was especially the case among women, who revealed a disc herniation risk of 3.2 (95% CI 1.6–6.3) in the highest category of cumulative lumbar load through intensive-load postures, but no elevated lumbar disc narrowing risk at all. However, in the control group materials handling is highly correlated with posture with a Kendall-Tau correlation coefficient of 0.61 for men and 0.78 for women. Therefore, collinearity could possibly explain the latter results.

When the analysis was restricted to lumbar spine load up to 10 years prior to diagnosis (lag-time analysis I), this had no substantial effect on the risk estimates for lumbar disc herniation or lumbar disc narrowing neither among men (table 2) nor among women (table 3). As past physical workload and current physical workload are highly correlated, we performed an additional subanalysis of all subjects that were not exposed to materials handling or intensive-load postures during 10 years prior to diagnosis (lag-time analysis II). Despite relatively low numbers, we revealed elevated odds ratios which approached statistical significance among men in the highest exposure category (OR 2.1, 95% CI 0.8 to 5.5 for lumbar disc herniation, and OR 2.8, 95% CI 0.9 to 8.8 for lumbar disc narrowing, respectively). Among women, the risk for lumbar disc diseases was highest in the second-highest exposure category (OR 1.9, 95% CI 0.9 to 4.3 for lumbar disc herniation, and OR 2.2, 95% CI 1.0 to 5.2 for lumbar disc narrowing, respectively).

## Discussion

In this study, we found a positive dose-response relationship between cumulative lumbar load and lumbar disc disease (disc herniation and disc narrowing) among men as well as among women. A distinction between the risks of manual materials handling and of intensive-load postures is difficult because of potential collinearity. Our lag-time subanalysis excluding subjects exposed during the last 10 years prior to diagnosis revealed elevated risks of lumbar disc diseases in men and women with high exposure to manual materials handling and working postures. These results point to a potential etiologic role of cumulative physical workload to lumbar disc disease even among persons with a long-term elimination of physical exposure. Strengths of our study include the extensive diagnostic procedure, the calculation of cumulative lumbar load based on expert assessment of occupational tasks and subsequent biomechanical analysis, and adjustment for multiple potential confounders according to an a-priori defined analytic concept.

As a potential limitation of the study, the low participation rate (66% among cases, 53% among control subjects) might have introduced selection bias. To further evaluate this potential bias, we asked non-participants by telephone about their longest held job. 57% of non-participating cases and 47% of non-participating control subjects gave their longest held occupation. According to this information, the proportion of blue-collar-workers was higher among non-participating cases and control subjects. Taking into account the higher response-rate among cases, the responder analysis does not point to substantial differential selection bias.

Due to lack of radiographic examination, the frequency of lumbar disc disease is unknown among the population controls. A suspected prevalence of lumbar disc disease of up to 10% among population controls would result in a slight tendency to underestimate potential risk factors.

Age – which is strongly associated with lumbar disc narrowing – was included in 10-years-categories, making residual confounding possible. When age – and additionally squared age – was included as a continuous variable in the regression model, this did not substantially alter the results. We therefore regard substantial residual confounding by age as improbable.

Persons with high physical workload might seek medical advice more frequently than persons with low physical workload. To investigate this potential detection bias, we performed two subanalyses: Firstly, we restricted the case group to patients with severe lumbar disc disease being accompanied with paralysis (no table, results of this subanalysis can be received by the authors). The results of this subanalysis did not substantially differ from the results of the main analysis. Secondly, we analysed the health seeking behavior of control subjects with low back pain (according to NORDIC questionnaire [21]). Among subjects with low back pain, the cumulative occupational lumbar load was not significantly associated with the cumulative incidence of medical consultation because of low back pain. Furthermore, among control subjects with low back pain, the conduction of X-rays of the lumbar spine was not significantly associated with physical workload; X-rays of the lumbar spine were undertaken in 70% of the heavily exposed subjects and 64% of the non-exposed subjects. According to these subanalyses, we regard a considerable detection bias as an improbable explanation of our study results. However, we cannot exclude the possibility that heavy physical workload might have led to the exacerbation of symptoms in the presence of a lumbar disc pathology that otherwise – without physical workload – might have remained clinically unapparent.

### Does a positive dose-response relationship exist between occupational lumbar load and lumbar disc diseases (lumbar disc herniation as well as lumbar disc narrowing)?

Several studies show an association between manual handling of objects and awkward working postures with the diagnosis of lumbar disc disease [1-16]. However, few studies have even considered a potential dose-response relationship between physical workload and lumbar disc disease. Braun [9] compared patients with surgically treated lumbar disc herniation with control subjects without complaints; he found a positive dose-response relationship between physical workload and disc herniation risk. In a case-control study, Kelsey et al. [13] revealed a positive association between lumbar disc herniation and lifting objects of more than 11.3 kg more than 25 times per day on average. Hofmann et al. [11] revealed an association between length of employment in occupations with high spinal load (e.g., nurses) and the risk of lumbar disc herniation. In an own (A.S., U.B., G.E.) case-control study a steep dose-response relation was shown between physical workload through carrying/lifting of weights (>5 kilograms) or extreme forward bending and symptomatic osteochondrosis/spondylosis [15] and lumbar disc herniation [16].

The present study reveals evidence for a positive dose-response relationship between manual materials handling as well as intensive-load postures and care seeking with the diagnosis of lumbar disc disease (lumbar disc herniation as well as lumbar disc narrowing) among men and women. In the highest dose category, we partly see an unchanged or even a decreased risk; this phenomenon can be found even among men when the highest dose category is further divided according to the 95-percentile of the distribution of control subjects (no table, results of this subanalysis can be received by the authors). The lack of a monotonous risk increase for very high cumulative lumbar load might be explained by a healthy worker survivor effect. However, we cannot exclude the possibility of effectively lacking further risk increase in highly exposed workers.

### Which type of exposure does contribute to the lumbar disease risk?

In accordance with the biomechanical concept of lumbar-spine force as the pathogenic pathway from physical workload to lumbar disc disease, our study deals with the influence of lumbar compressive force from materials handling and intensive-load postures on lumbar disc disease. According to our study, even moderate lumbar load might contribute to the risk of lumbar disc disease. Several experimental studies suggest that compressive forces can lead to structural changes in intervertebral discs (for example, decreased disc thickness) as well as to changes in intervertebral disc cell metabolism [27-30]. Hutton et al. [29] found an association between compressive force applied across the lumbar intervertebral discs of dogs for up to 27 weeks in vivo and quantitative changes in proteoglycans and collagen in the disc.

Because of the potential collinearity between object handling and posture, a definite conclusion concerning the separate effects of these exposures is not derivable. Exposure to manual handling of objects might play a role particularly in lumbar disc narrowing, while exposure to trunk inclination might be of particular importance for the development of lumbar disc herniation. This would be in accordance with Seidler et al. [15,16], who reveal a clear dose-response relationship between cumulative exposure to weight lifting/carrying and symptomatic osteochondrosis/spondylosis, whereas exposure to weight lifting and carrying was of limited relevance to isolated lumbar disc herniation. However, our exposure-specific results might alternatively be attributed to regression model instability through potential collinearity of object handling and posture. Therefore, we regard our basic approach of an exposure variable combining manual handling of objects and trunk inclination as reasonable.

### Does past physical work play an etiologic role in the development of lumbar disc disease (in other words: is a cumulative lumbar-load dose model appropriate)?

In keeping with the pathogenic concept of a long-term increased biomechanical load of lumbar-spine structures being an important cause of lumbar disc disease, the present study uses cumulative occupational exposure as the independent variable. When all exposures which occurred during 10 years prior to diagnosis were neglected, this did not substantially alter the results. A further subanalysis of all subjects that were not exposed to manual handling of objects or trunk inclination during 10 years prior to diagnosis revealed elevated risks of lumbar disc disease. This suggests that past physical work plays an etiologic role in the development of lumbar disc herniation as well as lumbar disc narrowing. Our finding is in accordance with Seidler et al. [15], who as well found an association between past physical workload and lumbar disc disease (symptomatic osteochondrosis/spondylosis).

## Conclusion

In conclusion, our results support a clear dose-response relationship between cumulative lumbar load and lumbar disc herniation as well as symptomatic lumbar disc narrowing.

## Competing interests

The authors declare that they have no competing interests.

## Authors' contributions

AS conceived the epidemiological analysis, performed the statistical analysis and drafted the manuscript, AB and JG developed the diagnostic procedure, MJ and AL performed the biomechanical lumbar-load analysis, RE and DD developed and coordinated the expert assessments of physical work, GE and FH participated in the interpretation of the study results, OL performed the verification of the clinical diagnosis as reference orthopedian, JH participated in the development of the epidemiological concept and in the interpretation of the study results, MM, GP, and BS developed the standardized interview and conducted plausibility checks, UB coordinated the study. All authors participated in the design and conduction of the study, read and approved the final manuscript.

## Appendix 1

Dose models applied in the German multi-center case-control study EPILIFT*

- *Dose models 1 and 2 *represent the cumulative lumbar-load dose model actually used in worker's compensation procedures in Germany ("Mainz-Dortmund Dose Model" [31]); they consider manual lifting or carrying of objects that involve a compressive force of 3.2 (in men) resp. 2.5 (in women) Kilo-Newtons or more at the lumbosacral disc; furthermore, lumbar load through trunk inclination of 90 degrees or more is considered. To calculate the daily cumulative lumbar load, the squared action-related compressive forces are multiplied by the corresponding task durations and summed up. Finally, this sum is multiplied with 8 hours – assumed as the common daily working duration – and the square root is calculated to receive dose values in Newtonhours. That means, the lumbosacral-disc compressive force is weighted overproportionally (squared as in all dose models 2 to 6) in relation to the respective duration of materials handling or intensive-load posture. All exposures with a daily cumulative lumbar load below 5,500 Newtonhours (in men) resp. 3,500 Newtonhours (in women) are neglected. While dose model 1 estimates compressive forces by few simple formulas, dose model 2 uses comprehensive three-dimensional dynamic biomechanical simulations.

- *Dose model 3 *is comparable to dose model 2, but considers (as all dose models 2 to 10) all modes of manual material handling (e.g., holding, pushing, pulling, shoveling), not only lifting and carrying. Compressive force is weighted analogously to dose models 1 and 2; the shift-dose thresholds for men and women are chosen equally to those of dose models 1 and 2.

- *Dose model 4 *provides the lowest exposure thresholds. For the calculation of cumulative lumbar load, in particular, manual handling of objects of about 5 kilograms or more and postures with trunk inclination of at least 20 degrees are considered. Lumbar-disc compressive force of 2.0 Kilo-Newtons or more is considered for day-related cumulation (as in all dose models 4 to 10) and is weighted analogously to dose models 1 to 3. No shift-dose thresholds are considered as in all dose models 4 to 10.

- *Dose model 5 *considers manual handlings of objects that involve a lumbosacral compressive force of 2 Kilo-Newton or more; furthermore, lumbar load through trunk inclination of 45 degrees or more is considered. Again, compressive force of at least 2.0 Kilo-Newtons is weighted overproportionally (squared) in relation to the respective action duration, i.e. analogously to dose models 1 to 4. No shift-dose thresholds are considered as in all dose models 4 to 10.

- *Dose model 6 *is comparable to dose model 5, but considers only lumbar load through trunk inclination of 75 degrees or more. No shift-dose thresholds are considered as in all dose models 4 to 10.

- *Dose models 7 to 10 *differ from dose model 5 only in the weighting of the compressive force in relation to the respective action duration. For example, in dose model 7 the lumbar-disc compressive force is equally weighted (linearly) as the respective action duration, and this weighting is varied (force to the power of 2, 3 and 4 in the dose models 8 to 10). No shift-dose thresholds are considered as in all dose models 4 and 10.

*Remark: There is no dose model that shows the best goodness of fit in all four case groups. Altogether, the dose models 4, 6 and 7 show the best goodness of fit.

## Pre-publication history

The pre-publication history for this paper can be accessed here:


